# Minimally Invasive Treatment Options for Hepatic Uveal Melanoma Metastases

**DOI:** 10.3390/diagnostics13111836

**Published:** 2023-05-24

**Authors:** Abin Sajan, Samuel Fordyce, Andrew Sideris, Connie Liou, Zeeshan Toor, John Filtes, Venkatesh Krishnasamy, Noor Ahmad, Stephen Reis, Sidney Brejt, Asad Baig, Shaheer Khan, Michael Caplan, David Sperling, Joshua Weintraub

**Affiliations:** 1 Department of Radiology, Columbia University Medical Center, 622 West 168th Street, New York, NY 10032, USA; 2 Department of Medicine, Columbia University Medical Center, 161 Fort Washington Avenue, New York, NY 10032, USA

**Keywords:** uveal melanoma, liver metastases, interventional radiology, locoregional therapy

## Abstract

Uveal melanoma is one of the most common primary intraocular malignancies that accounts for about 85% of all ocular melanomas. The pathophysiology of uveal melanoma is distinct from cutaneous melanoma and has separate tumor profiles. The management of uveal melanoma is largely dependent on the presence of metastases, which confers a poor prognosis with a one-year survival reaching only 15%. Although a better understanding of tumor biology has led to the development of novel pharmacologic agents, there is increasing demand for minimally invasive management of hepatic uveal melanoma metastases. Multiple studies have already summarized the systemic therapeutic options available for metastatic uveal melanoma. This review covers the current research for the most prevalent locoregional treatment options for metastatic uveal melanoma including percutaneous hepatic perfusion, immunoembolization, chemoembolization, thermal ablation, and radioembolization.

## 1. Introduction

Uveal Melanoma is a rare cancer accounting for about 3–5% of all melanoma cases in the United States [1]. It is the most common primary intraocular malignancy that accounts for about 85% of all ocular melanomas [2]. While melanoma traditionally originates from the basal layer of the epidermis, uveal melanoma originated from the melanocytes located along the uveal tract [3]. The pathophysiology and epidemiology between cutaneous and uveal melanoma are distinct and multiple studies have explored these differences.

The management of uveal melanoma is closely related to the tumor biology and its metastatic potential. The 5-year survival is approximately 80% and up to 50% of patients develop metastases, most commonly to the liver [4]. There has been limited success in the development of therapeutic agents that will limit the metastatic potential. Additionally, metastatic disease is associated with a poor prognosis with one-year survival only reaching about 15% [5]. 

Given the prevalence of metastatic disease and the associated poor prognosis in uveal melanoma, there is a need to identify treatment options that will decrease the tumor burden, affect the quality of life and improve overall survival. Multiple chemotherapeutic and immunological agents have been introduced in recent years with promising results [6]. Concurrently, advancements in minimally invasive technology have led to the growth of several minimally invasive treatment options for patients with uveal metastases. 

The purpose of this paper is to discuss the most prevalent locoregional treatment options for metastatic uveal melanoma including percutaneous hepatic perfusion, immunoembolization, chemoembolization, thermal ablation, and radioembolization (Table 1).

## 2. Percutaneous Hepatic Perfusion

Percutaneous hepatic perfusion (PHP), first reported in 1961 for the treatment of hepatic malignancies, delivers high concentrations of chemotherapeutic agents directly to the hepatic vasculature while limiting the systemic toxic side effects [12]. Before the evolution of isolated hepatic perfusion, patients underwent exploratory laparotomy to isolate the hepatic vasculature and relied on extracorporeal techniques for systemic circulation. The surgical approach was ultimately less successful due to the high rates of morbidity/mortality associated with such an invasive procedure and the limited number of repeat treatments since follow-up surgeries were likely not feasible or well tolerated [13]. In contrast, PHP is a minimally invasive option that utilizes a double balloon catheter in the inferior vena cava which filters hepatic venous drainage for chemotherapeutic agents before returning blood to the right atrium. 

**Figure 1:**
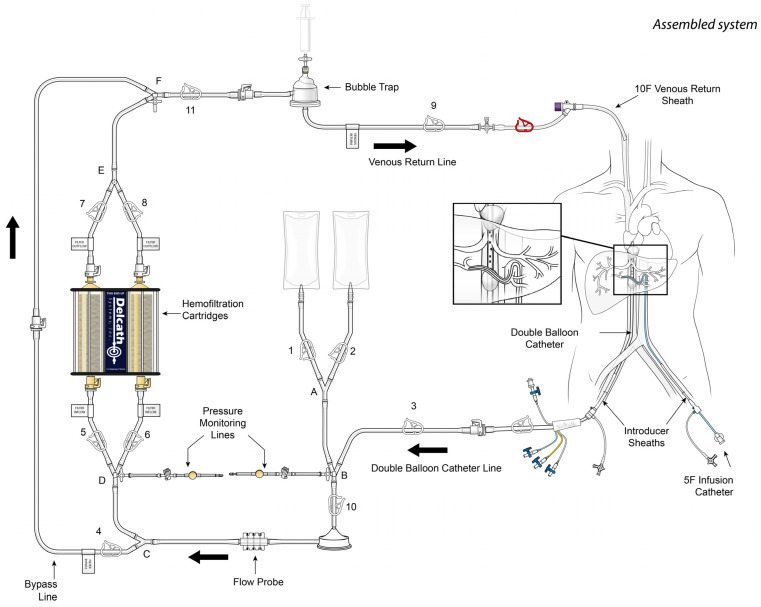
Illustration of the PHP system. With permission from Delcath Systems, Inc.

The procedure has been well described in the literature and requires general anesthesia and a dedicated extracorporeal perfusionist [14]. It requires three forms of access: right internal jugular venous access for blood return, common femoral venous access to isolate the hepatic venous outflow, and common femoral artery for access to the hepatic arteries for chemotherapeutic infusion (Figure 1). A hepatic angiogram is first performed to evaluate the hepatic vasculature and identify infusion locations. A double balloon catheter is then deployed in the inferior vena cava with one balloon directly below the right atrium and the other inferior to the hepatic veins but superior to the renal veins. After the hepatic vasculature has been isolated, the chemotherapeutic agent is infused at the predetermined arterial infusion points. Fenestrations within the double balloon catheter allow blood to be aspirated from the isolated hepatic venous vasculature and filtered through an extracorporeal circuit, which removes toxic chemotherapeutic agents prior to blood re-entering systemic circulation via the right internal jugular venous catheter.

The earliest data on the feasibility of PHP was first published in 2005 when a phase I dose escalation study using melphalan was conducted in 28 patients and demonstrated a 50% response rate in the uveal melanoma population subset based on the ‘Response Evaluation Criteria in Solid Tumors’ (RECIST) criteria [15]. Multiple retrospective studies have since been published supporting the use of PHP for metastatic uveal melanoma. Karydis et al. conducted a multicenter retrospective analysis of 51 patients with metastatic uveal melanoma treated with PHP/melphalan [16]. Partial or complete response was seen in patients as high as 43.1%. The hepatic progression-free survival was 9.1 months an overall median survival rate of 15.3 months. 

To date, a single muti-center randomized controlled trial has been conducted comparing PHP with melphalan (PHP-mel) with best alternative care (BAC). Hughes et al. randomized a total of 93 patients with liver predominant metastatic uveal melanoma to treatment with PHP-mel or BAC, including systemic chemotherapy, embolization, and supportive care, between 2006 and 2009 [7]. Hepatic progression-free survival, the primary endpoint, was 7 months for PHP-mel and 1.6 months for BAC (*p* < 0.0001) (10). Other endpoints were similarly statistically significant including overall progression-free survival, 5.4 months for PHP-mel and 1.6 months for BAC (*p* < 0.0001), and the hepatic objective response, 36.4% for PHP-mel and 2.0% for BAC (*p* < 0.001). Median overall survival however was not significantly different with 10.6 months in the PHP-mel group compared with 10.0 months in the BAC group. This was attributed to the crossover to the PHP-mel group. Another large randomized controlled trial by Zager et al. comparing PHP-mel vs. BAC (transarterial chemoembolization, pembrolizumab, ipilimumab and dacarbazine), with the PHP-mel group receiving up to 6 PHP treatment repeated every 6–8 weeks [17,18,19]. The primary outcome was the objective response rate as per the RECIST 1.1 criteria and early results demonstrated 35.2% for the PHP-mel group and 12.5% for the BAC group (*p* = 0.0154). The median progression-free survival was 9.03 months among PHP patients and 3.12 months among BAC patients (*p* = 0.0007). The median overall survival was 20.53 months among PHP patients and 14.06 months among BAC patients. While treatments have concluded, the outcome data continues to be collected. 

## 3. Immunoembolization

Immuno-embolization, specifically using the granulocyte-macrophage colony-stimulating factor (GM-CSF), has shown promise in treating regional disease within the liver and conferring “immunity” to extrahepatic disease through immune modulation [20]. Various factors, such as increased stimulation of antigen-presenting cells and improved antigen uptake may play a role in allowing for increased tumor clearance compared to more traditional embolization techniques [21]. 

Sato et al. conducted a phase I clinical trial to investigate the feasibility and efficacy of immunoembolization using GM-CSF for metastatic hepatic uveal melanoma, with a particular emphasis on evaluating dose-limiting toxicity (DLT) and maximum tolerated dose (MTD) [8,22]. Thirty-nine patients with metastatic uveal melanoma to the liver, involving less than 50% of the liver parenchyma, were included in the study. Patients were treated every four weeks with GM-CSF emulsified in ethiodized oil, followed by gelatin sponge particles. DLT was only seen at 1500 μg in one patient causing grade 3 abdominal pain leading to the patient dropping out of the study and being placed into hospice care. No other patients experienced DLT at this specific dose. Multiple patients received GM-CSF doses of 2000 μg with only asymptomatic grade 3 elevation in liver enzymes. Otherwise, MTD was not reached in the study. Of thirty-one assessable patients, two showed complete responses versus eight partial responses and ten stable disease states. Multivariate analysis suggested that higher GM-CSF doses (≥1500 μg) correlated to longer overall survival along with prolonged extrahepatic progression-free survival (4). 

Yamamoto et al. were one of the first to compare chemoembolization using 1,3-bis (2-chloroethyl1)-1-nitrosourea (BCNU) to immunoembolization (GM-CSF) [23]. Fifty-three patients with metastatic uveal melanoma involving less than 50% of the liver were included within the study. Univariate analysis demonstrated that high dose GM-CSF (≥1500 μg) resulted in increased overall survival compared to chemoembolization (20.4 months vs. 9.8 months, respectively, *p* = 0.05) (4). Valsecchi et al. conducted a similar comparison double-blinded study comparing immunoembolization with GM-CSF vs. bland embolization in 52 patients with biopsy proven metastatic uveal melanoma [8]. Partial responses (PRs) were recorded in five from the immunoembolization group (ORR, 21.2%), compared to three in the bland embolization group (ORR, 16.7%). Stable disease was seen in twelve patients in the immunoembolization group and nineteen in the bland embolization group. Immunoembolization correlated with delayed progression of extrahepatic systemic metastases. In patients with more than 20% liver involvement, OS was improved in the immunoembolization group. However, greater than 20% liver involvement and elevated lactate dehydrogenase (LDH) levels correlated with worse OS and PFS overall. Interestingly, it was also found that interleukin-6 (IL) 6 and IL-8 were significantly elevated in the bland embolization group after 18 h; however, the GM-CSF group experienced a much quicker increase in levels of ILs with the addition of TNF-alpha within 1 h after treatment (*p* < 0.001). Longer PFS was correlated with higher levels of TNF-alpha and IL-6 at 1 h (*p* < 0.001) and TNF-alpha at 18 h (*p* = 0.02) in the immunoembolization group. In the multivariate model, IL-8 levels at 18 h correlated with OS. Finally, there was no difference in adverse events between the two groups. 

Early results from immunoembolization with GM-CSF have been promising and have led to the evolution of potential combination therapies with systemic therapy [24]. Studies are currently underway to evaluate the efficacy of ipilimumab and nivolumab in combination with immuno-embolization for the treatment of metastatic uveal melanoma [25]. 

## 4. Transarterial Chemoembolization

Transarterial chemoembolization (TACE) is one of the most popular locoregional therapies available for liver tumors. The procedure involves lipiodol or particle embolization of the tumor along with the delivery of local chemotherapeutic agents. In conventional TACE (cTACE), chemotherapeutic agents such as doxorubicin, cisplatin, epirubicin, or mitomycin are mixed with Lipiodol, an ethiodized poppyseed oil that acts as a drug-delivering carrier as well as an embolic agent [26]. Other variations of drug delivery have evolved over the years such as drug-eluting beads (DEBs) which carry and release the chemical compounds in a controlled and standardized manner to reduce systemic toxicities [27]. A phase II randomized control study compared cTACE vs. DEB-TACE in the treatment of HCC and demonstrated that the DEB-TACE group was associated with higher rates of complete response, objective response, and disease control compared with the cTACE group (27% vs. 22%, 52% vs. 44%, and 63% vs. 52%, respectively) [28]. Most importantly, the DEB-TACE was associated with a significant reduction in free doxorubicin levels, serious liver toxicity and a lower rate of doxorubicin-related side effects including hepatic and cardiac disjunction [29]. 

Although the majority of TACE literature is focused on hepatocellular carcinoma, there is increasing focus on utilizing TACE for hepatic metastases such as uveal melanoma. 

Mavligit et al. published one of the earliest studies in 1988 with cisplatin and polyvinyl sponge chemoembolization for uveal melanoma metastasis [30]. The median survival range was about 31 months for responders and about 10 months for non-responders. Cisplatin has also been studied in combination treatments with Gupta et al. reporting cisplatin with or without hepatic artery infusion (HAI) therapy, which demonstrated a median overall survival of 15.8 months in responders versus 6.1 months in non-responders depending on the specific combination therapy [31]. The addition of HAI affected PFS but not OS. Tumor involvement of greater than 75% of the liver and elevated LDH levels correlated with a worse prognosis. 

3-bis (2-chloroethyl)-1-nitrosourea (BCNU) has also been used in the chemoembolization of uveal melanoma liver metastases [32]. The hepatic extraction rate of BCNU is 6 times higher than cisplatin. It is a lipophilic agent which allows it to be easily dissolved in lipiodol. The absence of Kupffer cells in melanoma metastases leads to a high regional concentration of ethiodized oil and thus BCNU [33]. Patel et al. used BCNU with an absorbable gelatin sponge and reported a median survival for complete/partial response of 21.9 months, stable disease of 8.7 months, and progressive disease of 3.3 months [9]. When stratified by percentage of hepatic involvement, there was a median survival of 19.0 months for <20% hepatic involvement, 5.6 months for 20–50% involvement, and 2.1 months for >50% involvement. Gonsalves et al. further explored the role of BCNU and enrolled patients specifically with either 50–75% or >75% tumor burden but used a higher dose of BCNU [33]. Although tumor sizes dramatically decreased after treatment, there was no significant difference in the overall survival between the different tumor burden groups.

Overall, these studies demonstrated that treatment response and survival were linked to the initial volume of the liver affected by metastatic disease. For example, in cases of <25% liver involvement, there can be a response rate of 33–86% with expected survival benefit in responders (Huppert et al.). Other laboratory values, including LDH, should be considered when selecting patients for chemoembolization. Patients with elevated LDH were shown in some studies to have a shorter time to progression and overall survival [31,34]. As expected, tumors that were hypervascular had better treatment responses [35]. Additionally, nodular discrete metastases had longer progression-free survival than infiltrative tumors [36]. 

The most common side effects included post-embolization syndrome with abdominal pain, nausea, vomiting, and fever as well as elevated liver enzymes. However, more severe complications did occur and included vascular thromboses, tumor lysis syndrome leading to acute kidney injury, and potentiation of liver dysfunction leading to liver failure. Many of these symptoms were often treated with intravenous hydration, pain medication, and prophylactic steroid treatment although the pre-treatment medications varied by study. One of the major toxicities specifically for BCNU is bone marrow toxicity leading to thrombocytopenia which can limit or delay further treatments. Additional studies are necessary to better understand the side effect profile of BCNU, especially in patients with multiple treatments. 

## 5. Percutaneous Thermal Ablation

Thermal ablation, both radiofrequency ablation (RFA) and microwave ablation (MA) are minimally invasive percutaneous image-guided techniques that cause thermally induced coagulation necrosis of the target tissue. RFA creates a closed-loop rapidly alternating electrical circuit which agitates ions and causes frictional heat transfer to the ablation site [37]. MA is a more recent technique, and the delivery of microwaves (900–2450 MHz) causes the rotation of water molecules (dipole rotation) and subsequent tissue agitation/frictional heat generation to the ablation site [38]. Unlike RFA, grounding pads are not required with MA. Both RFA and MA are associated with minimal adverse events with large multicenter studies citing rates of procedural complications as low as 1–2% [39,40]. 

A target temperature between 60–100 degrees Celsius is generally considered adequate to cause irreversible cellular damage. Temperature above this range increases the risk of incomplete ablation secondary to tissue vaporization and desiccation for RFA but not MA. Ultimately, the goal of thermal ablation is to achieve similar results as hepatic resection with a 360-degree 1 cm thick tumor-free ablation margin [41]. There are multiple significant differences between RFA and MA which are related to the distribution of thermal energy and the heat sink effect. The thermal energy is highest at the tip of the needle in RFA while the remaining ablation cavity receives thermal conduction of energy, leading to a heterogeneous distribution of heat [41,42,43]. Microwave energy is homogenously distributed through the target tissue and is less susceptible to target tissue conductive properties and nearby vasculature that causes a heat sink effect. 

Unlike RFA and MA, cryoablation (CA) is another percutaneous thermal ablation technique that uses the cooling of tissue to cause cell damage. The use of cryoablation in the liver was historically limited due to a poorly understood reported life-threatening complication of cryoshock, which causes multiorgan failure with high rates of mortality [44]. However, recent studies have questioned the prevalence of cryoshock with one study reporting a single episode in over 2000 cases when an appropriate limit to the targeted tumor volume has been set (upper limit of 5 cm) [44,45,46,47,48]. 

Thermal ablation in uveal melanoma hepatic metastases is limited to patients with oligometastatic disease. Alternatively, patients with less than three lesions can also seek surgical resection, but there is increasing interest in minimally invasive interventions for oligometastatic lesions [49,50]. Multiple retrospective case series have evaluated patient outcomes after thermal ablation for oligometastatic disease [51,52]. In a case series from 2013, eight patients with liver metastases who were treated with surgery and/or radiofrequency ablation had a median survival of 46 months when all lesions were treated [53]. A larger retrospective study of 44 patients comparing laparoscopic surgery/RFA vs. systemic therapy was published in 2015 (resection, *n* = 2, RFA, *n* = 14, systemic, *n* = 28) [54]. The median survival after diagnosis of liver metastases was 35 months in the surgery/RFA group compared to 15 months in the systemic therapy group (*p* < 0.0001). Additionally, five-year survival was 22% in the laparoscopic surgery/RFA group compared to zero in the systemic therapy group. Another 48-patient retrospective review, including 32 patients with liver resection and 16 patients with percutaneous ablation, demonstrated similar median OS between the two groups [10,54]. In 2016, a retrospective case series compared 72 patients who were treated with either tumor resection (*n* = 57) or RFA+/−resection (*n* = 15) [55]. There was no statistically significant difference in the median overall survival and disease-free survival between the groups.

As the technology of thermal ablation has advanced in recent years, there have been more novel applications of thermal ablation techniques. In 2019, Servois et al. investigated the success of RFA+/−resection in patients who previously had surgical resection with liver recurrence [56]. In a total of 14 patients, the recurrence-free survival was 56% two years after repeat treatment. The recurrence-free survival after repeat treatment was also similar in length to the recurrence-free interval after initial surgical resection (56% vs. 50% at 2 years). In 2020, Rozeman et al. studied the combination of RFA and immunotherapy (ipilimumab) for uveal melanoma hepatic metastasis [57]. Although the combination treatment did not demonstrate a significantly improved survival benefit compared to ipilimumab monotherapy, it opened the doors for further combination studies with immunotherapy agents. 

Unlike RFA, there is limited data studying the efficacy of MA or CA for uveal melanoma hepatic metastasis. Sterbis et al. published a small case series in 2020 of 13 patients with uveal melanoma hepatic metastases who underwent MA, oligometastatic or oligoprogressive [58]. The median overall time to progression was 8 months, and 10 of 13 patients were still alive with a median follow up time of 25 months. Shen et al. combined cryoablation with the transarterial infusion of pembrolizumab for uveal melanoma liver metastases [59]. In the combined stage, patients underwent subtotal cryoablation on day 1 followed by transarterial infusion of pembrolizumab on day 3 every three weeks. In the subsequent infusion stage, transarterial pembrolizumab infusion monotherapy was performed every three weeks. The median overall and hepatic progression-free survival times were 4.0 and 5.73 months, respectively. Although the median overall survival time was not reached, the estimated 6- and 12-month overall survival rates were 72.4% and 61.3%, respectively. 

## 6. Radioembolization

Liver metastases are predominantly supplied by arterial blood compared with the largely portal vascularization of normal hepatic parenchyma. Therefore, transarterial radiation doses can be applied to tumors while mostly sparing normal liver parenchyma by selectively delivering Yttrium-90 (90Y) labeled microspheres via the hepatic artery (radioembolization). The microspheres are predominantly β-emitters with a maximum soft-tissue penetration of 11 mm [60]. After the application of 90Y-labelled microspheres, response rates of hepatic metastases of up to 80 to 90% have been observed [61].

Due to the multifocality of hepatic metastases of uveal melanoma, the majority of patients have bilobar disease and are not candidates for surgical resection. Although radioembolization has long been an established and effective approach for primary and metastatic hepatic tumors, its use in metastatic uveal melanoma was not reported until a retrospective multicenter review in 2009 [62]. 11 patients receiving 12 treatments with a median activity of 1.55 GBq delivered per treatment demonstrated minimal toxicity, with PET/CT at 3 months posttreatment showing a complete response in one patient and a 77% response rate without evidence of radioembolization induced liver disease. 

Subsequent early studies solidified the role of radioembolization as a safe and effective salvage therapy for limited metastases from uveal melanoma. A 2011 study by Gonsalves et al. assessed the safety and efficacy of radioembolization in the management of patients with hepatic metastasis after failure of immunoembolization or chemoembolization [63]. The 32 patients within this single-institution study received a median activity of 1.08 GBq resulting in a median overall survival of 10.0 months and progression-free survival of hepatic metastasis of 4.7 months. Self-limiting grade 1–2 systemic toxicity included fatigue, indigestion, and abdominal discomfort. Grade 3–4 hepatic toxicity was attributed to tumor progression as evidenced by follow-up imaging. Patients who had a pre-treatment tumor burden of less than 25% had longer median overall survival (10.5 vs. 3.9 months) and progression-free survival (6.4 vs. 3.0 months) versus patients who had a pretreatment tumor burden of 25% or greater.

A 2012 retrospective analysis of thirteen patients treated with a radioembolization (mean activity of 1.78 GBq) as rescue treatment after failure of first-line chemotherapy and local therapies demonstrated partial response (PR) in 8 (62%), stable disease in 2 (15%), and progressive disease in 3 (23%) patients [64]. No significant treatment-related adverse events were reported. 

More recent publications have proposed radioembolization as the preferred first-line loco-regional therapy for uveal melanoma patients with hepatic metastases not suitable for surgery [65]. In a 2019 prospective phase II trial of radioembolization for the treatment of uveal melanoma hepatic metastases with less than 50% tumor burden, 23 treatment-naïve participants achieved median overall survival of 18.5 months with a 1-year survival of approximately 61% [11]. Additionally, 24 participants treated with radioembolization in whom prior immunoembolization treatment failed achieved median overall survival of 19.2 months with a 1-year survival of approximately 70%. Grade 3 treatment-related toxicities following radioembolization occurred in 3 of 23 treatment-naïve participants and in 1 of 24 participants in whom prior immunoembolization treatment failed.

Recent research has begun to explore the outcomes of multimodality therapies for treating hepatic metastases from uveal melanoma. Clinical data suggests radioembolization combined with immunotherapy is safe and effective [66]. Simultaneous immunotherapy at the time of 90Y therapy appears to be a predictor of prolonged overall survival and progression-free survival. In a 2020 retrospective review of 24 patients, those who received immunotherapy within 3 months of undergoing 90Y radioembolization demonstrated a median overall survival of 26.0 months versus 9.5 months for those undergoing Y90 radioembolization alone [67]. 

This apparent synergy between radioembolization and immunotherapy was investigated by Minor et al. in a phase II multicenter clinical trial. 26 patients with uveal melanoma hepatic metastases were treated with 90Y resin microspheres followed by immunotherapy with ipilimumab and nivolumab. The median progression-free survival was 5.5 months, and the median overall survival was 15.0 months. It is important to note that the initial dosing of 90Y and ipilimumab resulted in excessive toxicity. However, by limiting the background liver dose to 35 Gy and lowering the ipilimumab dose, toxicity was tolerable, and efficacy was not changed [68]. Radioembolization is felt to enhance the efficacy of immunotherapy by creating an immunogenic environment in the tumor [69]. 

## 7. Conclusions

In summary, there are multiple locoregional treatment options for patients with metastatic uveal melanoma, but the majority of these treatments have limited prospective data. More studies are needed to determine the optimal therapy for achieving maximum effectiveness while minimizing adverse events. Additional prospective randomized trials are necessary to determine the safety and effectiveness of combination locoregional and systemic therapies for the treatment of metastatic uveal melanoma. Although no current standardized treatment regimens exist for metastatic uveal melanoma, the data is encouraging, and future studies will help build a treatment algorithm. 

## Figures and Tables

**Table 1:** Summary of selected studies for each treatment group.

Study	Phase	*n*	Type of Therapy	Comparative Treatment	ORR (%)	Median hPFS (95% CI)	Median OS Months (95% CI)
Hughes et al. [7]	III	93	PHP-Mel	BAC	27.4%	5.4	10.6
Valsecchi et al. [8]	II	52	Immunoembolization with GM-CSF	Bland embolization	21.2%	3.9	21.5
Patel et al. [9]	II	24	BCNU-chemoembolization	None	16.7%	NA	5.2
Doussot et al. [10]	R *	48	Percutaneous ablation with liver resection	Liver resection	N/A	7.1	18
Gonsalves et al. [11]	II	24	Y-90 Radioembolization	None	NA	8.1	18.5

hPFS: hepatic progression-free survival; PHP-Mel: percutaneous hepatic perfusion with melphalan; BAC: best alternative care (embolization, systematic chemotherapy, and supportive care); GM-CSF: granulocyte-macrophage colony stimulating factor; BCNU: 1,3-bis(2-chloroethyl)-1-nitrosourea; * R: Retrospective.

## Data Availability

No new data were created or analyzed in this study. Data sharing is not applicable to this article.
